# Alteration to Dopaminergic Synapses Following Exposure to Perfluorooctane Sulfonate (PFOS), in Vitro and in Vivo

**DOI:** 10.3390/medsci4030013

**Published:** 2016-08-16

**Authors:** Rahul Patel, Joshua M. Bradner, Kristen A. Stout, William Michael Caudle

**Affiliations:** 1 Department of Environmental Health, Rollins School of Public Health, Emory University, Atlanta, GA 30322, USA; lefty1992@gmail.com (R.P.); joshua.m.bradner@emory.edu (J.M.B.); kristen.stout@emory.edu (K.A.S.); 2 Center for Neurodegenerative Disease, School of Medicine, Emory University, Atlanta, GA 30322, USA

**Keywords:** dopamine transporter, perfluorooctane sulfonate, striatum, tyrosine hydroxylase, vesicular monoamine transporter 2

## Abstract

Our understanding of the contribution exposure to environmental toxicants has on neurological disease continues to evolve. Of these, Parkinson’s disease (PD) has been shown to have a strong environmental component to its etiopathogenesis. However, work is still needed to identify and characterize environmental chemicals that could alter the expression and function of the nigrostriatal dopamine system. Of particular interest is the neurotoxicological effect of perfluorinated compounds, such as perfluorooctane sulfonate (PFOS), which has been demonstrated to alter aspects of dopamine signaling. Using in vitro approaches, we have elaborated these initial findings to demonstrate the neurotoxicity of PFOS to the SH-SY5Y neuroblastoma cell line and dopaminergic primary cultured neurons. Using an in vivo model, we did not observe a deficit to dopaminergic terminals in the striatum of mice exposed to 10 mg/kg PFOS for 14 days. However, subsequent exposure to the selective dopaminergic neurotoxin, 1-methyl-4-phenyl-1,2,3,6-tetrahydropyridine (MPTP) significantly reduced the expression of dopamine transporter (DAT) and tyrosine hydroxylase (TH), and resulted in an even greater reduction in DAT expression in animals previously exposed to PFOS. These findings suggest that PFOS is neurotoxic to the nigrostriatal dopamine circuit and this neurotoxicity could prime the dopamine terminal to more extensive damage following additional toxicological insults.

## 1. Introduction

As a greater number of chemicals are manufactured and used in a variety of industrial processes and consumer products, our appreciation for their impact on human health has also increased. In particular, the nervous system appears to be uniquely vulnerable to damage following exposure to environmental toxicants, especially highly halogenated compounds [1,2]. Indeed, extensive work from our group has characterized the neurological consequences of exposure to various organohalogen chemicals, including insecticides and industrial compounds [3,4,5,6,7,8,9,10,11,12].

More recently, attention has been directed towards another class of halogenated chemicals, perfluorinated compounds (PFCs), of which perfluorooctane sulfonate (PFOS) is a major constituent [13]. These compounds have been used extensively in manufacturing and consumer goods, including in fire-fighting foam, as lubricants, liquid and stain repellants in clothing, textiles, carpeting, and upholstery. The utility of PFOS in these media is attributed to their physicochemical properties, which directly contribute to its stability and resistance to breakdown and metabolism in both the environment as well as human body [14,15,16]. Indeed, significant levels of PFOS have been recorded in human tissue, including the blood and brain, suggesting a potential for neurological disruption [17,18,19,20]. Population-based examination of the contribution of PFOS exposure and neurological deficits is sparse. However, a recent study did identify a slight association between cord blood levels of perfluorinated compounds and reductions in newborn thyroid hormone, which is critical to normal neurodevelopment [21]. Although the manufacture and use of PFOS has been discontinued in favor of revised PFC compounds, the stability and bioaccumulative properties of these prior compounds allows them to remain in the environment and body tissue for extended periods of time, increasing the potential for human exposure and raising the risk of neurological damage [14,22,23].

Previous laboratory work has demonstrated the neurotoxicological effects of exposure to PFOS as well as other PFCs [24,25,26,27,28]. To date, the majority of studies have focused on the impact of exposure on pathological and behavioral deficits served by the hippocampus and frontal cortex. Alterations in spatial memory and locomotor activity have been identified with corresponding alterations in a variety of synaptic proteins involved in trophic support and intracellular signaling. Interestingly, findings in some of these studies identified alteration to select proteins involved in regulating dopamine signaling in the dopamine circuit. In particular, changes in the expression of postsynaptic dopamine receptors as well as the rate-limiting dopamine synthesis enzyme, tyrosine hydroxylase, was observed in the hippocampus and limbic system [24,27]. These findings are important as the dopamine system appears to be uniquely vulnerable to damage following exposure to environmental chemicals, many of which have been implicated as risk factors for the neurodegenerative disorder, Parkinson’s disease (PD) [5,29]. PD is characterized by extensive damage to the nigrostriatal dopamine circuit, resulting in significant loss of dopamine neurons in the substantia nigra pars compacta (SNpc) and subsequent reduction of dopamine innervation and dopamine concentrations in the striatum [30]. Previous work from our group has demonstrated the neurotoxicological impact of environmental chemicals on the dopamine system, highlighting the vulnerability of the presynaptic terminal in the striatum to damage [4,6,7,8,9,11,12,31,32].

Given our current understanding of the role exposure to environmental chemicals plays in the etiopathogenesis of PD, it is critical to extend upon these findings and further characterize the neurotoxicological effects of emerging environmental chemicals on the nigrostriatal dopamine circuit. With these ideas in mind we designed this study to specifically assess the effect of PFOS exposure on the nigrostriatal dopamine pathway through the coupling of in vitro and in vivo models. Through these approaches we found PFOS to be selectively neurotoxic to dopamine neurons, in vitro. These findings were extended, in vivo, using a subacute exposure paradigm that allowed us to evaluate PFOS neurotoxicity using adult male mice. Although exposure to PFOS did not elicit overt damage to the presynaptic dopamine terminal in the striatum, damage was exacerbated following challenge with the selective dopamine neurotoxin, methyl-4-phenyl-1,2,3,6-tetrahydropyridine (MPTP). Moreover, this potentiation may be due to the ability of PFOS to inhibit the sequestration of dopamine into synaptic vesicles. These findings suggest that PFOS can preferentially damage the nigrostriatal dopamine system and, more importantly, increase the vulnerability of the dopaminergic synapse to additional toxicological insults through alteration of dopamine handling.

## 2. Materials and Methods

### 2.1. Chemicals and Reagents

Perfluorooctane sulfonate (PFOS) was purchased from Sigma-Aldrich (St. Louis, MO, USA). HEK293 and SH-SY5Y cells were obtained from American Type Culture Collection (ATCC; Manassas, VA, USA). Hibernate A and Hibernate A-Calcium were purchased from BrainBits (Springfield, IL, USA). B27, DNase1, and Neurobasal A were purchased from Life Technologies (Life Technologies, Carlsbad, CA, USA). Papain was obtained from Sigma-Aldrich. Dispase II was purchased from Roche (Nutley, NJ, USA). Aphidicolin was purchased from A.G. Scientific (San Diego, CA, USA). Whatman GF/F filter papers were obtained from Brandel, Inc. (Plantation, FL, USA). The bicinchoninic acid (BCA) protein assay kit was obtained from Pierce (Rockford, IL, USA). 1-methyl-4-phenyl-1,2,3,6-tetrahydropyridine (MPTP) was purchased from Sigma-Aldrich. Monoclonal anti-rat dopamine transporter (DAT) and polyclonal anti-rabbit tyrosine hydroxylase (TH) antibodies were purchased from EMD Millipore (Billerica, MA, USA) or Pel Freez Biologicals (Rogers, AR, USA). Polyclonal anti-rabbit gamma-aminobutyric acid (GABA) transporter 1 (GAT1) and vesicular glutamate transporter (vGlut) antibodies were purchased from Synaptic Systems (Gottingen, Germany). Monoclonal anti-mouse α-tubulin antibodies were purchased from Sigma-Aldrich. Mouse anti-microtubule associated protein 2 (MAP2) antibodies were purchased from Abcam (San Francisco, CA, USA). Secondary antibodies conjugated to horseradish peroxidase were obtained from Jackson Immunoresearch Laboratories (West Grove, PA, USA). Secondary antibodies conjugated to fluorescent tags were obtained from Life Technologies. SuperSignal West Dura extended duration substrate and stripping buffer were obtained from Pierce. 3,3′ Diaminobenzidine (DAB) was purchased from Sigma-Aldrich. The lactate dehydrogenase (LDH) Assay Kit was obtained from Cayman Chemicals (Ann Arbor, MI, USA). ^3^H-Dopamine (^3^H-DA) was purchased from Perkin-Elmer (Boston, MA, USA). Cold tetrabenazine was obtained from Sigma-Aldrich.

### 2.2. Culturing of SH-SY5Y Cells

SH-SY5Y cells were originally derived from the SK-N-SH neuroblastoma cell line, which was isolated from human bone marrow. These cells were chosen for these experiments given their general neuronal phenotype and dopaminergic properties, including enzymes necessary for dopamine synthesis and metabolism, as well as dopamine handling. Cells were cultured in Dulbecco’s Modified Eagle Medium F12 (DMEM/F12) supplemented with 100 units/mL penicillin and 100 units/mL streptomycin and 10% fetal bovine serum (FBS). Cells were cultured at 37 °C in a humidified atmosphere with 5% CO_2_ and propagated according to the protocol provided by the supplier. When cells were confluent, they were trypsinized and collected for passage to working concentrations in 96-well culture plates for treatment with PFOS.

### 2.3. Culturing of HEK-hVMAT2 Cells

HEK293 cells stably expressing human- vesicular monoamine transporter 2 (VMAT2) constructs were cultured at 37 °C and 5% CO_2_ in DMEM with 10% FBS [4]. Constructs were made in pcDNA3.1 and contained a zeocin resistance gene. Plasmids were transfected into HEK293 cells with Lipofectamine 2000. Stable cell lines were generated by repetitive rounds of limiting dilutions in selection media. The successful integration of hVMAT2 was confirmed by immunoblotting for VMAT2.

### 2.4. Cell Viability

Cell death was assessed using the LDH cell viability assay per the standard procedures provided by the supplier and as previously published [4]. Briefly, cells were passaged to 96-well culture plates and incubated in increasing amounts of PFOS for 24 h. Cell media was transferred to a new 96-well plate and LDH reaction solution was added to each well and incubated for 30 min at 37 °C. Absorbance was read on a plate reader at 490 nm and reported as average LDH release for each concentration.

### 2.5. Vesicular ^3^H-DA Uptake in HEK-hVMAT2 Cells

Cells grown to confluency were harvested, centrifuged at 1000× *g* (Eppendorf 5424/5424R rotor) and resuspended in 0.32 M sucrose, 5 mM 4-(2-hydroxyethyl)-1-piperazineethanesulfonic acid) (HEPES), at pH 7.4 with 1× Protease Inhibitor Cocktail [4]. The cell suspensions were centrifuged at 8000× *g* and the supernatant was removed and added to VMAT2 uptake buffer (2 mM ATP-Mg^2+^, 1.7 mM ascorbate, 25 mM HEPES, 100 mM potassium tartrate, 0.1 mM ethylenediaminetetraacetic acid (EDTA), 0.05 mM ethylene glycol-bis(β-aminoethyl ether)-N,N,N′,N′-tetraacetic acid (EGTA), pH 7.4), or uptake buffer containing 200 µM tetrabenazine (TBZ). Cell fractions were incubated with increasing amounts of PFOS dissolved in dimethyl sulfoxide (DMSO) or control solutions lacking PFOS, followed by the addition of a 10 mM dopamine stock with a 6% ^3^H-DA tracer and incubated for 5 min. Reactions were terminated and samples harvested with a Brandel Cell Harvester with Whatman GF/F Filter Paper. Bound activity on membranes was measured using a scintillation counter and specific activity was determined by subtracting total disintegrations per minute (DPMs) from TBZ-sensitive (total-nonspecific) DPMs and normalized to the total protein per well as determined by BCA protein assay.

### 2.6. Primary Culture of Mesencephalic Neurons

Briefly, ventral mesencephalic neuron cultures were prepared from postnatal mice (postnatal day 1–3) as previously published by our group [4,12]. Mouse brains were dissected in ice cold Hibernate A supplemented with B27. Following isolation of the relevant region and the removal of meninges, tissue pieces were chemically treated with a dissociation solution containing Papain (1 mg/mL), Dispase II (1.2 U/mL), and DNase 1 (1 μL/mL) dissolved in Hibernate A- Calcium for 20 min at 37 °C and gently agitated every 5 min. Tissue was then rinsed in plating media containing Neurobasal-A medium, 10% heat inactivated FBS, penicillin-streptomycin, and mechanically dissociated using gentle trituration. Cells were plated on poly-d-lysine pre-coated 96-well plates at 40,000 cells per well. Plating media were removed and immediately switched to Neurobasal-A-based culture media containing B27, 1% l-glutamine and 1% penicillin-streptomycin after 2 h, in vitro. The following day, culture media containing aphidicolin (1 μg/mL) was added to reduce the proliferation of glial cells in culture. Approximately one half of the culture media from each well was replaced every four days. Primary cultures were treated on day 8 in vitro with five concentrations of PFOS dissolved in cell culture media. After 24 h, cells were fixed in 4% paraformaldehyde (PFA) for 20 min and incubated overnight in rabbit anti-TH and mouse anti-MAP2 at 4 °C. The following day, cultures were incubated with fluorescent secondary antibodies, goat anti-rabbit 488 and goat anti-mouse 572 for 1 hr at room temperature. After staining with DAPI, cells were rinsed and stored in phosphate buffer saline (PBS). Images of treated cultures were obtained using an Array Scan VTI HCS (Cellomics; Pittsburgh, PA, USA). Forty-nine contiguous fields were taken per well and TH+ neurons were counted and analyzed using GraphPad analysis software (La Jolla, CA, USA).

### 2.7. Animals and Treatment

Eight-week-old C57BL/6J male mice were purchased from Charles River Laboratories (Wilmington, MA, USA) and allowed to acclimate to housing for seven days before initiation of treatment. Mice were orally gavaged with 25 µL of 10 mg/kg of PFOS dissolved in a corn oil vehicle daily for 14 days, similar to previously published reports [4,6,12]. This dosing paradigm was intended to represent the primary route of human exposure to PFOS. Two cohorts of mice were used for this study. Cohort 1 was exposed to PFOS (*n* = 6) or control (*n* = 6) for 14 days and then sacrificed 24 h after the last dose. Cohort 2 was similarly exposed to PFOS (*n* = 6) or control (*n* = 6) and then 24 h following the last dose, mice were administered the dopaminergic neurotoxin, MPTP. MPTP was injected subcutaneously at 10 mg/kg at 7:00 a.m. and 7:00 p.m. and then animals were allowed to sit for seven days before they were sacrificed and brain tissue collected for subsequent experimental assays [11]. Exposure to PFOS or MPTP did not elicit overt changes in general health outcomes or activity levels of the mice. Animals were evaluated daily for reductions in mobility that would limit their access to food and water and were weighed every two days in order to ensure that they were not losing weight due to the PFOS or MPTP treatments. Animals were also observed for grooming and interactions with cage mates to ensure maintenance of social engagement and lack of aggression. Standard rodent chow and tap water were available ad libitum. All procedures were conducted in accordance with the Guide for Care and Use of Laboratory Animals (National Institutes of Health) and have been approved by the Institutional Animal Care and Use Committee at Emory University.

### 2.8. Western Blot Analysis

Western blots were used to quantify the amount of DAT, TH, GAT1, vGlut, and α-tubulin present in samples of striatal tissue from treated and control mice. Analysis was performed as previously described [4,6,11,12,33]. Briefly, striata samples were homogenized and samples subjected to polyacrylamide gel electrophoresis and electrophoretically transferred to polyvinylidene difluoride membranes. Nonspecific sites were blocked in 7.5% nonfat dry milk in Tris-buffered saline, and then membranes were incubated overnight in a monoclonal antibody to the *N*-terminus of DAT. DAT antibody binding was detected using a goat anti-rat horseradish peroxidase secondary antibody (1:10,000) and enhanced chemiluminescence. The luminescence signal was captured on an Alpha Innotech Fluorochem imaging system (Hampton, NH, USA)and stored as a digital image. Membranes were stripped for 15 min at room temperature with Pierce Stripping Buffer and sequentially reprobed with α-tubulin (1:1000), TH (1:1000), GAT1 (1:2500), and vGlut (1:10,000) antibodies. α-Tubulin blots were used to ensure equal protein loading across samples.

### 2.9. Immunohistochemistry

Tissue staining was performed as described previously [33]. Briefly, brains were immersion fixed in 4% PFA and serially sectioned at 40 μm. Sections were incubated with a monoclonal anti-DAT or polyclonal anti-TH antibody overnight and then incubated in a biotinylated goat anti-rat or rabbit secondary antibody for 1 h at room temperature. Visualization was performed using 3,3′-DAB for 3 min at room temperature. After DAB, all sections were dehydrated and coverslipped. Bright field images were captured at 5× magnification using a Zeiss Axio Imager M2 microscope (Thornwood, NY, USA).

### 2.10. Statistical Analysis

Statistical analysis of the effects of PFOS on SH-SY5Y cells and primary cultured neurons was performed on raw data for each treatment group by one-way ANOVA. Analysis of the effects of PFOS on dopaminergic and non-dopaminergic endpoints by immunoblotting was performed on raw data from each treatment group by Student’s *t*-test or one-way ANOVA. Post hoc analysis was performed using Tukey’s post hoc test. Significance is reported at the *p* < 0.05 level.

## 3. Results

**Figure 1:**
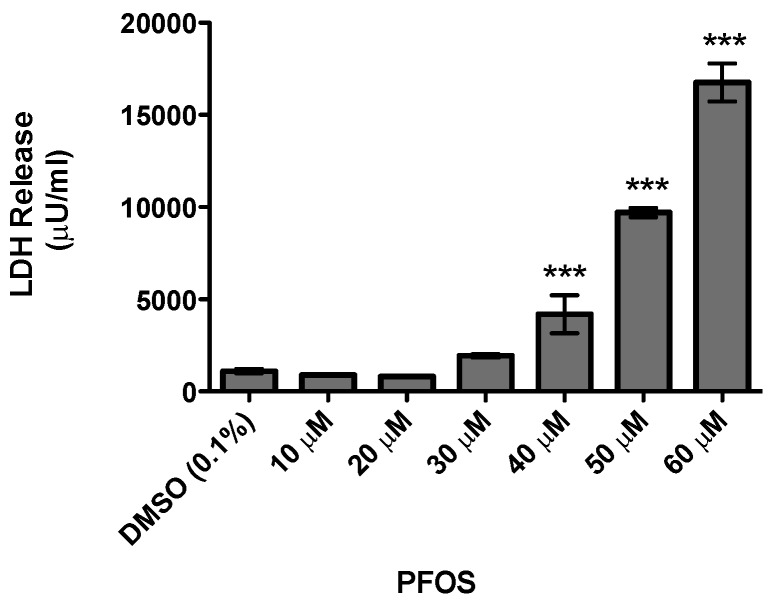
Acute exposure to perfluoroctane sulfonate (PFOS) is cytotoxic to undifferentiated SH-SY5Y dopaminergic neuroblastoma cells, as measured by lactate dehydrogenase (LDH). Beginning at 40 μM, PFOS treatment resulted in a statistically significant increase in toxicity, compared with dimethylsulfoxide (DMSO) treated cells. The amount of toxicity continued to increase with elevated concentrations of PFOS. Columns represent the means of raw data + SEM of eight experimental replicates per treatment group performed over three separate experiments. *** Values statistically significant from DMSO control (*p* < 0.001).

As the major focus of this study was to evaluate the neurotoxicity of PFOS on the dopamine circuit, we initially characterized the general cytotoxicity of PFOS using the undifferentiated SH-SY5Y neuroblastoma cell line, which has been used extensively as an in vitro model in PD research [34]. Cells were treated for 24 h with DMSO or increasing concentrations of PFOS and then cytotoxicity was determined via the release of LDH. Exposure to lower concentrations of PFOS did not elicit a measurable elevation in LDH release. However, beginning at 40 μM, a significant elevation in LDH release was observed with increasing concentrations of PFOS (Figure 1). These findings demonstrate the neurotoxicity of PFOS in a dopaminergic cell line and provided critical dosing information for subsequent in vitro assessment of dopaminergic neurotoxicity by PFOS.

**Figure 2:**
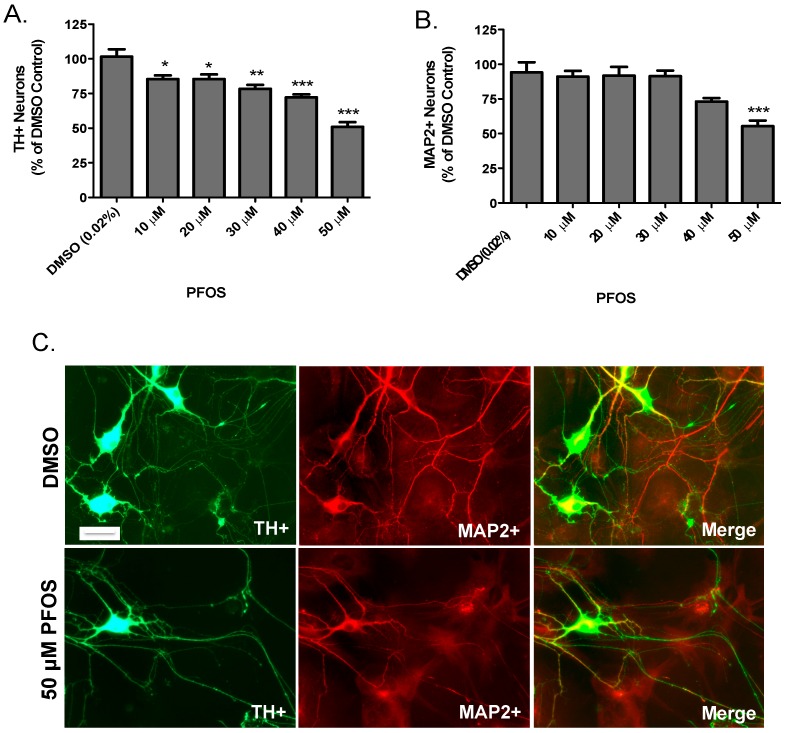
Acute exposure to PFOS results in a preferential reduction in the number of primary cultured dopaminergic neurons. (**A**) Treatment with PFOS resulted in a significant reduction in the number of TH+ neurons, beginning at 10 μM; (**B**) treatment with PFOS did not elicit overt loss of non-dopaminergic neurons (MAP+), except at the highest concentration of PFOS (50 μM); and (**C**) representative images of TH+ and MAP2+ neurons following treatment with DMSO or 50 μM PFOS. Scale bar: 10 μm. Columns represent the percent change from DMSO control. Data represent the mean + SEM of 6 experimental replicates per treatment group performed over three separate experiments. * Values statistically significant from DMSO control (*p* < 0.05). ** Values statistically significant from DMSO control (*p* < 0.01). *** Values statistically significant from DMSO control (*p* < 0.001).

Extending our SH-SY5Y data, we then evaluated the dopaminergic effects of PFOS on primary cultured neurons isolated from the dopamine-rich ventral mesencephalon, which is a major target of dopaminergic neurodegeneration in PD. Neurons were exposed to DMSO or increasing concentrations of PFOS for 24 h before they were fixed and quantified for loss of dopaminergic and non-dopaminergic neurons. Using TH as a selective marker for dopamine neurons, PFOS exposure elicited significant reductions in TH-positive neurons at each concentration of PFOS tested (Figure 2A). In contrast, PFOS did not appear to have a measurable effect on non-dopaminergic neurons, except at the highest concentration used (Figure 2B). Representative images in Figure 2C demonstrate the significant reduction in dopaminergic and non-dopaminergic neurons at the highest concentration of PFOS. Taken in sum, these findings suggest that dopaminergic neurons are selectively vulnerable to PFOS-induced toxicity, compared with non-dopaminergic neurons.

**Figure 3:**
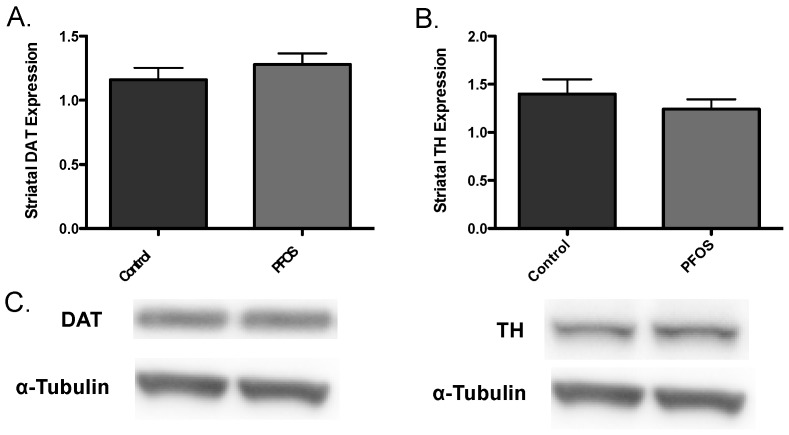
In vivo exposure to PFOS does not elicit an alteration in synaptic dopaminergic proteins. Exposure of nine-week-old C57BL/6J male mice to 10 mg/kg PFOS for 14 days did not result in an explicit alteration in the expression levels of striatal DAT (**A**) or TH (**B**). (**C**) are representative immunoblots of DAT and TH. α-Tubulin was included to ensure even protein loading. Columns represent the mean of raw values + SEM (six mice per treatment group).

**Figure 4:**
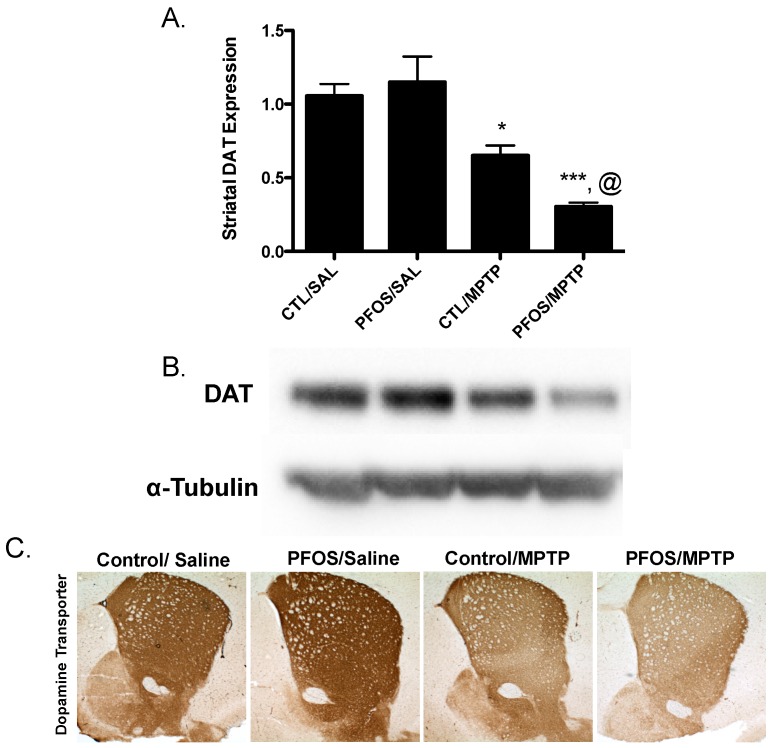
Challenge with the dopamine neurotoxin, MPTP, causes significant reductions in DAT expression following PFOS treatment. An additional cohort of nine-week-old C57BL/6J male mice were exposed to 10 mg/kg PFOS for 14 days. One day following the last PFOS exposure, mice were injected with saline or 10 mg/kg MPTP at 7:00 a.m. and 7:00 p.m. and then allowed to sit for seven days before analysis. (**A**) Treatment with MPTP resulted in a significant reduction in the expression in striatal DAT in control animals that did not receive PFOS. Moreover, treatment with MPTP exacerbated reductions in striatal DAT in animals that had received PFOS prior to MPTP treatment; (**B**) and (**C**) are representative immunoblot and immunohistochemical processing, respectively, demonstrating the reductions in DAT expression in the striatum of treated animals. α-Tubulin was included to ensure even protein loading. Columns represent means of raw data + SEM (six mice per treatment group). * Values statistically significant from CTL/SAL (*p* < 0.05). *** Values statistically significant from PFOS/SAL (*p* < 0.001). @ Values statistically significant from CTL/MPTP (p < 0.01).

**Figure 5:**
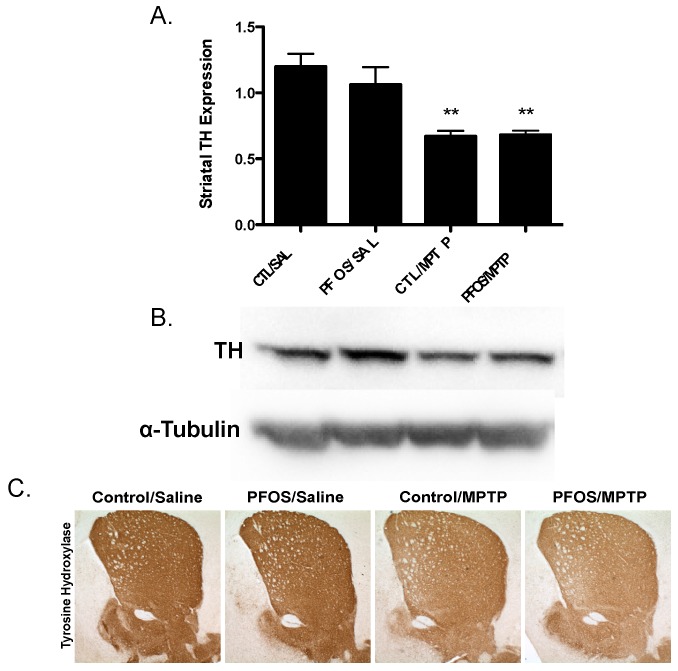
Challenge with the dopamine neurotoxin, MPTP, does not cause significant reductions in TH expression following PFOS treatment. (**A**)Treatment with MPTP elicited a significant reduction in the expression of striatal TH in control animals. However, prior exposure to PFOS did not potentiate a greater reduction in TH following MPTP treatment. (**B**) and (**C**) are representative immunoblot and immunohistochemical processing, respectively, demonstrating the reductions in TH expression in the striatum of treated animals. α-Tubulin was included to ensure even protein loading. Columns represent means of raw data + SEM (six mice per treatment group). ** Values statistically significant from CTL/SAL or PFOS/SAL (*p* < 0.01).

**Figure 6:**
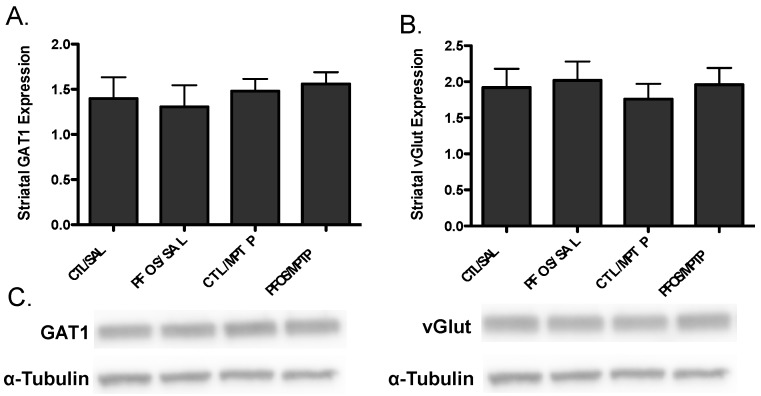
PFOS and MPTP do not alter the expression of GABAergic or glutamatergic proteins in the striatum. Neurotoxicological effects of PFOS appear to be preferential for dopaminergic terminals, as no change was observed in expression of GABAergic (**A**) or glutamatergic (**B**) presynaptic proteins in the striatum; and (**C**) representative imunoblots for vGAT and vGlut. α-Tubulin was included to ensure even protein loading. Columns represent means of raw data + SEM (six mice per treatment group).

Drawing from our in vitro data, we further elaborated our assessment of PFOS on the nigrostriatal dopamine circuit using an in vivo platform. C57BL/6J male mice were exposed via oral gavage to 10 mg/kg PFOS, daily, for 14 days and then evaluated for alterations in expression of key presynaptic dopamine proteins in the striatum, known to change in PD. Interestingly, exposure to PFOS did not elicit any measureable deficits in either DAT or TH (Figure 3). Previous work from our group has demonstrated that exposure to various environmental neurotoxicants can exacerbate damage to the dopamine system following a challenge with another neurotoxin [8,9,11]. With this in mind, we examined whether challenge with the selective dopaminergic neurotoxin, MPTP, could unmask underlying damage to the dopamine terminals that was not previously identified. Indeed, similar to results in Figure 3, exposure to PFOS did not cause changes in expression of DAT or TH. However, when PFOS-treated animals were challenged with MPTP an exaggerated reduction in DAT expression was observed, compared with animals that received corn oil and MPTP (Figure 4). While MPTP definitively reduced the expression of TH in the striatum, a combination of PFOS and MPTP did not appear to cause a further reduction (Figure 5). These findings appear to be selective for dopamine neurons as no change was observed in GABAergic or glutamatergic markers in the striatum (Figure 6).

**Figure 7:**
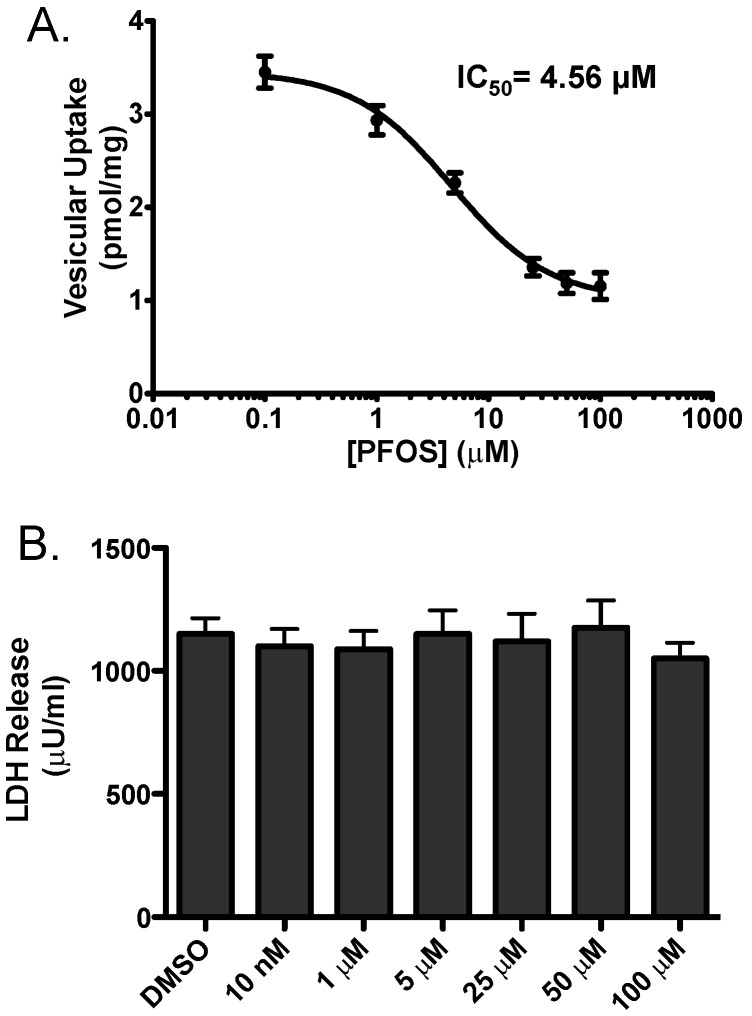
(**A**) ^3^H-dopamine uptake in HEK-hVMAT2 cells following exposure to PFOS caused a dose-dependent inhibition of dopamine transport through VMAT2. Fractions of HEK-hVMAT2 cells were incubated with PFOS or DMSO followed by incubation with ^3^H-dopamine. Resultant radioactive uptake into vesicles is shown. Results demonstrate PFOS inhibits dopamine uptake by VMAT2 with an IC_50_ = 4.56 Μm; (**B**) Reduction in dopamine uptake by VMAT2 was not due to PFOS-induced cytotoxicity of HEK-hVMAT2 cells, as assessed by LDH release. HEK-hVMAT2 cells were incubated for 10 min in increasing concentrations of PFOS at similar concentrations used for uptake. Each experiment was performed in triplicate.

In order to address the potential mechanistic underpinnings of these findings we employed an in vitro model to evaluate the effects of PFOS on inhibition of vesicular dopamine uptake. HEK cells engineered to stably express VMAT2 (HEK-hVMAT2) were acutely treated with increasing concentrations of PFOS and then assessed for the ability of VMAT2 to sequester ^3^H-dopamine. With this approach we found PFOS effectively inhibits VMAT2 with an IC_50_ of 4.56 μM. To confirm that these reductions in VMAT2 function were not due to cytotoxicity, we treated HEK-hVMAT2 cells with similar concentrations of PFOS for 10 min and found not change in LDH release at any of the concentrations tested. These findings suggest that inhibition of VMAT2 function was not due to cytotoxicity of the cells (Figure 7).

## 4. Discussion

The nigrostriatal dopamine system has been shown to be extensively vulnerable to damage by a variety of environmental toxicants, highlighting exposure to these chemicals as critical risk factors in PD [5,29]. The majority of work to elucidate these chemical culprits has focused on highly halogenated compounds, including pesticides and compounds used in industrial processes. More recently, attention has turned towards the neurological impacts elicited by PFCs. With an increasing understanding of the neurotoxicity of these compounds, our knowledge of the effects these chemicals have on the dopamine system remains immature. With these gaps in mind, we coupled an in vitro and in vivo platform to specifically address the neurotoxicity of PFOS on the nigrostriatal dopamine system.

Our initial approach to characterizing the neurotoxicity of PFOS on dopaminergic cells was to employ an acute exposure to the SH-SY5Y neuroblastoma cell line, which resulted in significant cytotoxicity at the micromolar range. Establishing a generalized understanding of the neurotoxicity of PFOS was critical, as it served to guide our subsequent exposure paradigm in a more biologically complex model, to further assess the impact of PFOS on the nigrostriatal dopamine circuit.

Primary cultured neurons isolated from the ventral mesencephalon provide a powerful model to efficiently evaluate the neurotoxicity of chemicals on select neuronal populations and can assist in further refining neuronal targets for investigation, in vivo. Using this model, our findings demonstrate a preferential neurotoxicity of PFOS to dopaminergic neurons at low micromolar concentrations that was not seen in the non-dopaminergic population, predominantly composed of GABAergic cells. To our knowledge, this is the first evidence of PFOS-induced dopaminergic neurotoxicity using primary cultured neurons. The explanation for the preferential damage of the dopaminergic neurons to PFOS could be due to the ability of PFOS to generate reactive oxygen species coupled with the innate vulnerability of dopaminergic neurons to oxidative stress [35,36]. Indeed, in both SH-SY5Y cells as well as cerebellar granule cultures, exposure to PFOS has been shown to generate significant levels of reactive oxygen species [37,38,39], which were attenuated by increasing the antioxidant, glutathione, through supplementation with *N*-acetyl cysteine.

If PFOS selectively damaged nigrostriatal dopamine neurons, in vitro, we surmised that exposure to PFOS would have similar effects, in vivo, potentially via alteration of presynaptic machinery that is critical to proper dopamine handling. To evaluate this hypothesis we employed a subacute exposure paradigm of 10 mg/kg PFOS for 14 days. As our understanding of the neurotoxicological impacts of PFOS and how these relate to the body’s burdens in the human population is still being elaborated, we based our current treatment regimen on our previously published studies that have evaluated similar highly halogenated chemicals in adult mice [3,4,6,12]. Although not evaluated in this study, previous animal studies that have employed a similar dosing paradigm have found concentrations of PFOS in plasma to be approximately 130 μg/mL [40]. These levels are significantly higher than those previously observed in the serum of human subjects, which have been measured near 0.023 μg/mL [41]. In the future, analysis of serum concentrations of PFOS in experimental animals will allow for an enriched understanding of how exposure concentrations equate to body burden, allowing for alignment of these exposures with values identified in the human population. However, the concentrations used in this study are in line with more recent studies that identified alterations in dopaminergic receptors in the limbic system of mice treated with up to 6.0 mg/kg PFOS for 28 days [24]. In contrast to our original thinking, our findings demonstrate that exposure to PFOS using our subacute paradigm, did not elicit overt alterations to the presynaptic dopamine terminals in the striatum. However, previous work from our group has shown exacerbation of dopaminergic neurotoxicity following an additional toxicological challenge, effectively “unmasking” an underlying perturbation [42]. Thus, following exposure to PFOS, animals were subsequently exposed to the selective dopamine neurotoxin, MPTP. Preferential damage to dopamine neurons is determined by the selective uptake of its neurotoxic metabolite, MPP+ into dopamine neurons, via DAT, followed by inhibition of mitochondrial complex I, and the generation of reactive oxygen species [43]. Indeed, while treatment with MPTP significantly reduced the expression of DAT and TH in our study, prior exposure to PFOS appeared to “prime” the dopamine neurons to further toxic insult, resulting in an even greater reduction in DAT, but no change in TH. Interestingly, no effect was seen on GABAergic or glutamatergic proteins, similar to our results, in vitro. While our reductions in protein expression suggest a pathological alteration to the synaptic integrity of the dopamine terminal, further investigation is necessary to determine if these reductions equate to functional losses in these synaptic proteins. Coupling expression and functional alterations will provide a more informed understanding of the pathology that is occurring in this system. Reductions in DAT, but not TH, are not surprising, given previous work from our group that identified DAT expression as a critical marker of early damage to the dopaminergic terminals, following exposure to environmental toxicants or alterations in dopamine homeostasis [4,6,12,33,44]. Thus, DAT may serve as a sensitive cellular indicator of dopaminergic damage that precedes subsequent loss of dopamine terminal integrity, as defined by TH expression. The relevance of these findings is corroborated by imaging studies in patients with PD, who demonstrate a significant reduction in DAT expression prior to a clinical syndrome [45,46,47,48].

In order to address the possible underlying mechanistic processes that could contribute to the unmasking of dopaminergic terminal damage following exposure to MPTP, we focused on the function of VMAT2, which functions to sequester cytosolic dopamine into synaptic vesicles and prepare them for release at the plasma membrane during neurotransmission. Using an in vitro assay that allows us to assess the function of VMAT2 to transport dopamine into vesicles, we found that PFOS significantly inhibits VMAT2 function at low micromolar concentrations. Packaging of free dopamine into vesicles is a crucial part of maintaining not only normal dopamine homeostasis, but also the health of the dopamine neuron. Indeed, the inability to sequester dopamine into vesicles through loss of VMAT2 function has been shown to be neurotoxic to dopamine neurons through the generation of reactive species [33,44]. Our group as well as others has also shown VMAT2 to be a potential target for some environmental toxicants, including polychlorinated biphenyls, polybrominated diphenyl ethers, and hexabromocyclododecane, through reduction in its expression and function [4,6,12,49,50,51].

In addition to packaging dopamine, VMAT2 is also able to package MPP+ into vesicles, in turn, preventing it from being transported to the mitochondria and disrupting ATP synthesis. Indeed, MPP+ neurotoxicity to the dopamine system is predicated on the expression and function of VMAT2 [52,53,54]. Taken in concert, if PFOS was able to inhibit VMAT2 function in our in vivo model, it can be speculated that this also inhibited VMAT2 sequestration of MPP+, resulting in excess amounts of MPP+ in the cytosol and a greater potential to disrupt the mitochondria and generate oxidative stress. Although we relied on a selective dopaminergic neurotoxin to induce dopaminergic damage, these findings have greater implications for damage to the nigrostriatal dopamine system following exposure to multiple neurotoxicants or other trauma that could affect VMAT2 expression and function.

## 5. Conclusions

In conclusion, in this study we have coupled in vitro and in vivo approaches in an effort to evaluate the neurotoxicological effects of PFOS on the nigrostriatal dopamine system. These findings build upon previous work and serve to extend these findings by highlighting the vulnerability of the dopaminergic synapse to PFOS exposure and identifying a potential target in VMAT2, whose function may serve as a critical mediator of PFOS neurotoxicity. Moreover, these findings draw attention to PFOS as a potential risk factor for damage to the nigtostriatal dopamine circuit, which is damaged in PD, as well as other neurological diseases and disorders.
